# Earlier Nutrient Fortification of Breastmilk Fed LBW Infants Improves Jaundice Related Outcomes

**DOI:** 10.3390/nu12072116

**Published:** 2020-07-17

**Authors:** Xiao Wei Ma, Wei Qi Fan

**Affiliations:** 1 Faculty of Medicine, Dentistry and Health Sciences, The University of Melbourne, Grattan Street, Melbourne, VIC 3010, Australia; amanda.ma@nhw.org.au; 2 Department of Paediatrics, The Northern Hospital, 185 Cooper Street, Epping, VIC 3076, Australia

**Keywords:** jaundice, hyperbilirubinemia, LBW, preterm, fortification

## Abstract

This study aimed to evaluate jaundice outcomes of low-birthweight premature infants commenced on earlier versus later nutrient supplementation (80 mL/kg/day vs. 160 mL/kg/day; total fluid intake, F80 vs. F160). Demographics, feeding regimens, and clinical outcomes data were collected. Infant and maternal characteristics were similar. Earlier nutrient supplementation was associated with multiple improved jaundice outcomes: total (TSBR), unconjugated and conjugated (CSBR) serum bilirubin values (196 ± 46 vs. 228 ± 52, 184 ± 44 vs. 212 ± 50, 12 ± 4 vs. 16 ± 5, respectively, all *p* < 0.001); phototherapy (39% vs. 64%, *p* < 0.0001). % CSBR/TSBR ratio was similar between groups. For those on phototherapy, duration and median irradiance were similar. F80 infants experienced reduced: feeding intolerance (26.0% vs. 45.2%, *p* = 0.007); length of stay (16.0 ± 0.64 vs. 18.8 ± 0.74 days, *p* = 0.03), maximum weight loss as % birth weight (5% vs. 6%, *p* = 0.03); decrease in weight Z-score at 10 days (−0.70 ± 0.03 vs. −0.79 ± 0.03, *p* = 0.01). F80 infants regained birthweight earlier (10.0 ± 0.3 days vs. 11.5 ± 0.3 days, *p* < 0.0001) and had no differences in adverse clinical outcomes. We speculate that earlier nutrient supplementation improved jaundice outcomes due to enhanced excretion/elimination of bilirubin.

## 1. Introduction

The basic etiology of neonatal hyperbilirubinemia is well understood. Neonatal jaundice results from an imbalance between bilirubin production soon after birth and elimination. Factors include: bilirubin formation from the breakdown of heme present in normal red blood cells; conjugation in the liver with glucuronic acid to form a water-soluble form; excretion in the bile [1]; some hydrolysis of bilirubin glucuronide in the small intestine with resultant reabsorption of bilirubin; final removal via faeces [2].

What is not so well understood, despite decades of research, are the influences contributing to hyperbilirubinemia—which, in the neonate, is a multifactorial process, associated with both biochemical and physiological immaturity. In the near term to term infant, risk factors for developing severe unconjugated hyperbilirubinemia include: haemolytic disease; gestational age 35–36 weeks; cephalohematoma; exclusive breastfeeding, particularly if nursing is problematic and weight loss significant; East Asian race; macrosomic infants of a diabetic mothers; male gender [3]. On the other hand, a decreased risk of significant jaundice has been associated with: gestational age of 41 weeks; discharge from hospital after 72 h; exclusive bottle feeding [3].

An interrelationship between enteral feeding, the timing of feeds, nutrition and neonatal jaundice has long been recognised. In the 1960s, when 48 h fasting of newborn infants was common, studies showed that babies fed glucose in part saline or water at 4 h of life developed significantly less jaundice than fasting babies [4,5]. During the 1980s, it was noted that breastfed babies developed more jaundice than formula-fed babies [6,7]. An important factor in this finding is that breastmilk is rich in in β-glucuronidase, which, in the intestine, decouples glucuronic acid from conjugated bilirubin and allows bilirubin to be reabsorbed by the enterohepatic circulation [8]. At the turn of the century, further investigations revealed that a formula’s composition was a factor—infants on casein-hydrolysate formula developed less jaundice than those fed standard formula, which in turn was associated with less jaundice than breastfed babies [8]. The key role of the intestine in feeding was shown by a prospective randomised study where infants on parental nutrition developed less jaundice if they also had early hypocaloric enteral feeds [9]. In the near term to term infant, early or later parenteral nutrition has no effect on jaundice outcomes—indicating that the timing of nutrition is only a factor with enteral feeding [10].

In our Special Care Nursery (SCN), we manage mostly moderate (32 to 34 weeks’ gestation) to late (34 to 36 weeks’ gestation), low birthweight preterm infants. Previously, we commenced the fortification of expressed breast milk (EBM) or preterm formula at a full enteral feed achievement of 160 mL/kg/day, before moving to a protocol of earlier fortification/preterm formula at 80 mL/kg/day. In a prospective, observational study, we investigated the nutritional requirements of moderate and late preterm infants—an area which has been little researched [11]. We reviewed the available evidence on appropriate protein intake levels and showed that a daily protein intake of above 3 g/kg, an essential level to maintain fetal growth rates, could be provided soon after birth via the fortification of breast milk (delivering 1 g of protein per 100 mL of milk)—if commenced at an enteral volume of 80 mL/kg/day. This earlier protein boost was associated with other benefits such as decreasing the duration of post-birth weight loss and the incidence of feeding intolerance.

During the study, we found evidence to suggest a benefit of earlier nutrient supplementation at 80 mL/kg/day on jaundice outcomes—however, due to small sample size, our results did not achieve significance [11].

The aim of this study is to explore the link between an earlier commencement of fortification of enteral feeds and the evaluation of a possible improvement in jaundice outcomes. We hoped to achieve this by retrospectively examining the records of a sufficiently large cohort of infants during the period when the earlier protocol of commencing fortification at 160 mL/kg/day was in place, with a similar sized cohort following the protocol change that commenced fortification at 80 mL/kg/day.

## 2. Materials and Methods

### 2.1. Population Overview

This study’s participants were moderately preterm and late preterm LBW neonates admitted to the SCN between January 2012 to December 2018 at The Northern Hospital (TNH) in Epping, an outer suburb of Melbourne, Australia. 

The catchment area for TNH is a lower-socioeconomic, multicultural, multi-ethnic community with associated concerns such as obesity. 

### 2.2. Participation 

We performed a retrospective chart review. Assignment into F80 or F160 groups was done on the basis of chronology. Before 2016, our feeding protocol introduced breastmilk fortification once full enteral feeds had been achieved (160 mL/kg/day). Following this date, we changed our protocol to commence fortification at 80 mL/kg/day. No other changes were made to the protocol. Otherwise, the management of F80 and F160 infants was identical.

Inclusion criteria were: birthweight less than 2500 g; gestational age 31 weeks to late preterm; tolerating a total enteral fluid intake of less than 80 mL/kg/day. Infants with gastrointestinal malformations or recognized chromosomal abnormalities were excluded. Infants who did not strictly fit into the commencement criteria of 80 or 160 mL/kg/day enteral fluid intake were also excluded. Ethics approval was provided through the Northern Health Human Research Ethics Committee. (ALR No: ALR 52.2018).

### 2.3. Data Collection

Maternal baseline data collected include age, gravity and para, region of birth based on United Nations country grouping, and primary language spoken at home. Maternal clinical data collected include antenatal complications such as gestational diabetes, preeclampsia, and Group B Streptococcus screening result.

Baseline data collected on neonates include gestational age, sex, reason for prematurity, mode of delivery, 5-min APGAR score, and birthweight. Data on feeding regimens collected included: day of life at first enteral feed; total fluid intake at first enteral feed; total fluid intake at commencement of enteral supplementation; day of life at commencement of enteral supplementation. F80 neonates were defined as those that were commenced on human milk fortifier (or preterm formula if EBM was unavailable) at total fluid intake of 80 mL/kg/day, while F160 were defined as those that commenced on human milk fortifier (or preterm formula) at total fluid intake of 160 mL/kg/day.

### 2.4. Outcomes

The primary outcome was related to hyperbilirubinemia and included: need for phototherapy; maximum number of phototherapy lights required; highest recorded bilirubin value; number of days that phototherapy was required. Anthropometric outcomes included maximum weight loss, days to regain birth weight, and discharge weight. Z-scores were calculated for birthweight, lowest weight post birth and discharge weight using the 2013 Fenton growth charts [12]. Adverse clinical outcomes were presumed sepsis, hypoglycaemia, respiratory distress syndrome, seizures, necrotising enterocolitis and intraventricular haemorrhage. Feeding intolerance was present if neonates had frequently large volume vomits or large gastric residuals with clinical abdominal distention, if neonates required cessation of enteral feeds, and if there was radiological evidence of abdominal distention. Sepsis was presumed if there were systemic symptoms such as respiratory distress, fever, lethargy, and increased inflammatory markers such as C-reactive protein levels and full blood examination. Small for gestational age (SGA) was defined as weight being below the 10th percentile for gestational age using appropriate Fenton growth charts. Length of stay was defined as number of days admitted to the SCN.

The use of 2013 Fenton growth charts in this study requires an explanation. Fenton growth charts are based on intrauterine data as opposed to birthweight data used for the more recently developed and internationally widely accepted Intergrowth-21st growth charts. The issue of which type of growth chart is most appropriate has become controversial. While for extremely low birth weight infants, Intergrowth 21st Project growth standards have been shown to be superior to Fenton Growth Charts [13], the picture for moderate to late term infants is not so clear. A recent study, where more than 50% of infants were between 34 and 36 weeks of gestation, showed that for the 2-week period immediately following birth, Fenton growth charts were superior to Intergrowth 21st [14]. Of particular concern is that Intergrowth-21st charts underestimate the rate of SGA compared to Fenton charts, which is of relevance in a lower socioeconomic community such as is the case with our study [15]. In our own state of Victoria, a recent statewide survey of 28000 births concluded that “intrauterine charts appear to be the most sensitive in the detection of SGA infants at increased risk of adverse perinatal outcomes” [16]. On evaluation, we opted to remain with the 2013 Fenton growth charts as we are currently using these on a day to day basis in our SCN and our previously published study [11] used 2013 Fenton growth charts—thus allowing a valid comparison of results with this study. Clearly, any future study will need to evaluate the increasing acceptance and benefits of the Intergrowth 21st charts.

### 2.5. Feeding Protocol

Due to a policy of actively promoting and supporting breastfeeding, the majority of premature babies in our SCN are fed breastmilk. All study infants commenced feeding from day 1 at an enteral volume of 60 mL/kg and increased by 20 mL/kg daily as tolerated. The fortification/supplementation regime was standard and straightforward. Human milk fortifier (FM85, Nestle, Vevey, Switzerland) was introduced into expressed breastmilk (EBM) or preterm formula was commenced if EBM was not available (Pre-nan, Nestle, Vevey, Switzerland) or S26 low birth weight Formula (Aspen Nutritionals Pty Ltd., Clayville, South Africa). FM85 was given at the recommended concentration of 1 g per 20 mL of EBM which boosted the protein content of EBM by 1 g of protein per 100 mL. Preterm formula provided a protein content of 2.9 g/100 mL. Prior to 2016, such fortification was commenced at an enteral volume achievement of 160 mL/kg/day (F160 group). Post 2016, due to a policy change, fortification was commenced once enteral volumes had reached 80 mL/kg/day (F80 group). Fortification or preterm formula was given to all babies up to a weight of 2500 g. Thereafter, cessation was at the discretion of the neonatal consultant and, based on such issues as appropriate weight gaining, extent of feeding intolerance and SGA [11].

### 2.6. Data Analysis

Results were compared between F80 and F160 infants and differences in categorical variables were assessed using the Chi-square test or Fisher’s exact test where appropriate. Normality for continuous variables was assessed using the Shapiro-Wilk test, with *p*-value. Continuous variables were analysed using the student’s *t*-test or the Mann—Whitney test where appropriate. A *p* value of <0.05 was considered significant. Statistical analyses were performed using either SPSS (IBM Corp. Released 2017. IBM SPSS Statistics for Windows, Version 25.0. IBM Corp, Armonk, NY, USA) or NCSS 12 statistical programme (NCSS 12 Statistical Software (2018). NCSS, LLC. Kaysville, Utah, USA)

### 2.7. Sample Size Calculation

Assuming an 8% change (as indicated from the raw data of our previous study [11]) in the primary outcomes for the F80 group when compared with the F160 group, an alpha of 0.5 and power of 80%, a sample size of 93 was indicated in each group. 

## 3. Results

### 3.1. Overall Characteristics

A total of 215 babies were included in this study, 105 in the F80 group and 110 the F160 group. 

Infant gestational age ranged from 31 to 36 weeks. Between the F80 group and F160 group, there were no significant differences in birth weight, birth weight Z-scores, APGAR score at 5 min, reason for prematurity and mode of delivery (see Table 1).

Maternal age ranged from 16 to 44 years of age. Approximately half the mothers were born in Australia and New Zealand, with South Asia as the next largest place of maternal birth. English was the predominant language spoken at home. There were no significant differences in maternal characteristics between F80 and F160 groups (see Table 2).

### 3.2. Anthropometric Findings

The F80 group experienced less maximum weight loss as a percentage of birth weight compared to the F160 group. Infants commenced on earlier enteral supplementation regained birth weight before infants commenced on later enteral supplementation. Weight gain at 10 days as measured by change in Z-score from birthweight showed a significantly lower decrease in weight percentiles for F80 infants compared to F160 infants (see Table 3).

### 3.3. Jaundice Outcomes 

**Figure 1:**
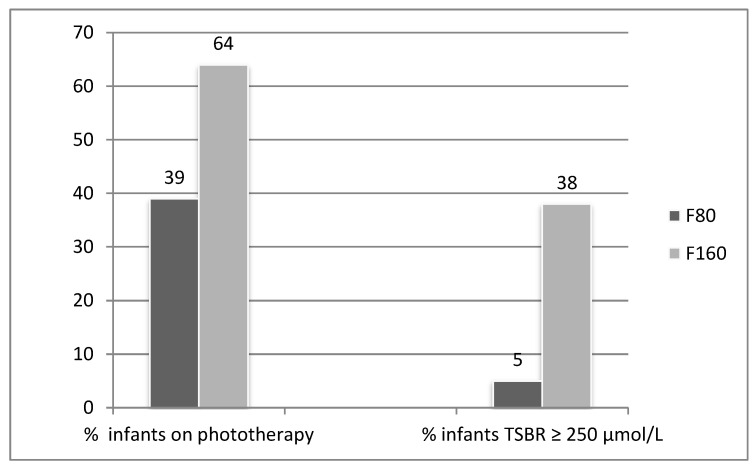
Proportion of infants requiring phototherapy and maximum value of TSBR (total serum bilirubin) level ≥ 250 µmol/L. *p* < 0.0001 for both categories, Chi-square test for probability.

Multiple positive jaundice outcomes were associated with earlier supplementation of fortified breastmilk or preterm formula. For the F80 group compared with the F160 group, these outcomes significantly included lower total serum bilirubin (TSBR), unconjugated bilirubin (USBR) and conjugated bilirubin (CSBR): less infants below a phototherapy recommended cut off value for LBW infants at 36 weeks gestation of TSBR 250 µmol/L [17]; fewer infants requiring phototherapy and therefore reduced irradiance as a cohort. For those on phototherapy, there were no differences between the number of phototherapy days required for each group and the median dose of irradiation was the same; see Figure 1 and Table 4.

### 3.4. Clinical Outcomes

Feeding intolerance was lower for the F80 group. Length of stay (LOS) was significantly shorter for F80 infants than F160 infants. There were no differences in the frequencies of presumed sepsis, hypoglycaemia and respiratory distress syndrome. There was no occurrence of NEC (see Table 5).

## 4. Discussion

As a consequence of changing our feeding protocol to an earlier commencement of fortification (at 80 mL/kg/day rather than 160 mL/kg/day), infants began supplementation of their feeds almost 5 days earlier—on average before day 3. The key finding of our study is that earlier nutrient supplementation of predominantly breastmilk fed babies (mainly by fortification) was associated with multiple beneficial jaundice outcomes such as lower TSBR, USBR and CSBR and lower phototherapy rates and consequently less exposure irradiation. As we have previously reported in some detail [11], although the nutrition requirements of moderate and late preterm infants is not well understood, earlier nutrition in the form of breastmilk fortification is well tolerated and not associated with any negative outcomes. 

It could be argued that these outcomes are not surprising when viewed against the changes to infant feeding regimes that have occurred over the last half century. One reason for the abandonment of the customary 48 h fasting of newborns in the 1960s was the realization that early hypocaloric feeding, such as glucose, reduced hyperbilirubinemia [5]. This is true even in the case of predominantly parenteral fed infants [9]. Early and frequent breastfeeds have shown to be even more effective in reducing hyperbilirubemia [18]. The evidence is mounting as to why earlier nutrition has a positive impact on neonatal jaundice. 

There is evidence to suggest that the expression of extrahepatic UDP-glucuronosyltransferase 1A1 (UGT1A1), may play such a role. UGT1A1 is solely responsible for bilirubin conjugation and had been thought to be only present in the liver [19]. In the neonate, liver conjugation of bilirubin is immature [20,21]. Recent evidence suggests that UGT1A1 is expressed in the intestine and contributes to bilirubin metabolism and elimination [22]. The regulation of intestinal UGT1A1 is by food derived nutrients—breastmilk contains substances that inhibit expression, whereas formula components enhance expression [23]. Is it possible that the improved jaundice outcomes observed in the F80 group resulted from enhanced conjugation of bilirubin due to the earlier exposure to fortifier components that switch on UGT1A1 expression in the neonatal intestine? 

At a basic level, neonatal jaundice can be thought of as an imbalance in the production and elimination of bilirubin [1]. The production—conjugation ratio (the percentage of CSBR to TSBR) has been shown to be useful in understanding the mechanism of hyperbilirubinemia. The ratio is inversely proportional to TSBR levels indicating that a balance or equilibrium exists between production and conjugation and can be used to compare the level of conjugation between subjects [24]. Our study showed that following a feeding protocol change from nutrient supplementation commencement at 160 mL/kg/day to 80 mL/kg/day, the approximately 5 days earlier commencement of supplementation in the F80 group was associated with significantly lower TSBR, CSBR and USBR. However, the CSBR/TSBR ratio was similar, with no significant difference suggesting that the effect of earlier nutrient supplementation did not alter the production—conjugation dynamics. These results are consistent with a hypothesis that liver conjugation via UGT1A1 has not been altered, but leaves open the possibility that intestinal UGT1A1 expression has occurred. This is because enterocytes containing UGT1A1 have the ability to re-conjugate intestinal sourced bilirubin that has been de-conjugated by breastmilk derived β-glucuronidase [8] and excrete directly to the intestinal lumen, without re-entering the enterohepatic circulation [25].

Oral glucose has been shown to be an effective inducer of intestinal UGT1A1 without affecting liver UGT1A1 induction and has been suggested as a convenient way to treat neonatal jaundice while continuing the benefits of breastfeeding [26]. The fortifier used in our SCN is Nestle FM-85 which has the starch maltodextrin as the major carbohydrate [27]. Maltodextrins are converted to glucose in the alimentary tract [28]. We therefore speculate that the earlier introduction of glucose via fortifier in the F80 infants (approximately 5 days before the F160 group—see Table 1) has led to intestinal UGT1A1 induction, with enhanced intestinal excretion of CSBR due to localized re-conjugation of bilirubin previously de-conjugated by the action of β-glucuronidase.

Zinc is another constituent of nutrient supplement that has been demonstrated to improve bilirubin elimination. A recent randomized, double blind trial demonstrated that neonates between 31 and 36 weeks gestation requiring phototherapy, had significantly reduced jaundice within 48 h of receiving oral zinc [29]. These findings followed previous animal studies which showed that zinc supplements reduced TSBR [30]. Zinc has been shown to increase bowel movements and therefore bilirubin fecal excretion, which in turn reduces the enterohepatic circulation of bilirubin [29]. We speculate that the earlier supplementation of zinc from fortification may also have allowed to the F80 group to manage bilirubin excretion more efficiently. 

Another possible excretion mechanism for bilirubin is bacterial conversion. In adults, bilirubin excreted into the intestinal lumen is very efficiently converted to urobilinogen in the faeces—a natural detoxifying mechanism which aids elimination [31]. In our study, peak jaundice levels occurred around day 4 to 5 after birth (Table 4). However, in healthy breastfed newborns, urobilinogens are not even detected until day 5 and are at very low levels compared to faecal content at 6 weeks of age [31]—suggesting that this mechanism is not likely to have played an important role in our study. Fecal fat has also been flagged as a mechanism for bilirubin elimination. Breastfed neonates have highly efficient reabsorption of faecal fat [32], which raises the possibility that nutrient supplement or preterm formula fat may not be as efficiently reabsorbed, thus trapping fat soluble bilirubin with a consequential decrease in TSBR [33]. This has not been shown to be the case. In breastfed or formula fed near term infants, no difference was found in fecal fat excretion rates or stool production [33].

Some recent studies have associated vitamin D deficiency in neonates with hyperbilirubinemia. TSBR significantly declined in vitamin D treated infants on phototherapy compared to those on phototherapy alone [34]. Neonates with out of physiological range TSBR have been shown to be vitamin D deficient compared to controls [35]. Low maternal vitamin D levels have been associated with these findings [36]. The mechanism behind this association is unknown, but it is speculated that as both bilirubin and vitamin D are metabolized in the liver, there may be an interaction exacerbating hyperbilirubinemia [35]. We do not think this a likely outcome in our study, as although nutrient supplements contain vitamin D, mothers were on routine vitamin D supplements during pregnancy. 

While the focus of this paper has been on the apparent benefits to jaundice outcomes due to earlier nutrient supplementation resulting from a change in our feeding protocol that commenced supplementation at 80 mL/kg/day rather than 160 mL/kg/day, there were other clear benefits. F80 infants regained birth weight earlier, weight Z-scores were better at 10 days, the incidence of feeding intolerance was less, and LOS was almost 3 days shorter with important cost saving implication for the hospital. There was no difference in discharge weights between either group. This finding is consistent with findings that while phototherapy itself may result in a temporary weight reduction, catch up growth occurs with eventually no weight difference to infants not exposed to irradiation [37]. The reduction in feeding intolerance with earlier nutrient supplementation, is similar to our earlier finding [11]. The reason for this beneficial reduction in the inability to digest enteral feedings is not clear, but may be related to the earlier introduction of micronutrients coinciding with a threshold point in development of the immature and disorganized preterm intestinal tract [11]. One such micronutrient may be zinc, which has a trophic effect on intestinal mucosa, modulates intestinal permeability and has a role in influencing intestinal microbiota composition [38,39]. 

A key limitation of this study is that being retrospective in nature, it has inferior evidence when compared to a prospective study. The convenience sampling inherent in this type of retrospective study may have led to selection bias. Of particular note is the concern of temporal relationships. This study stretched over a 6-year period, yet only a total of 215 infants were included in the study. The reason for this was the strict adherence to the criteria of only including infants commenced on fortification at 80 or 160 mL/kg/day—infants who commenced fortification at any other volume, eg 70 or 150 mL/kg/day were excluded. While this strict selection criteria improved the reliability of outcomes (particularly when compared to our previous prospective observational study [11]), it lengthened the period of the study in order to acquire a sample size above the calculated 93 cases for each group. Consequently, over this period, difficult to assess factors may have had some impact on the findings. 

Another potential limitation, as discussed in Methods, is that this study used Fenton 2013 growth charts to classify infants as SGA and to determine weight Z-scores. While this allowed a direct comparison with our previous study [11] and intrauterine based growth charts are still used extensively in Australia, Intergrowth 21st charts are being used in many other parts of the world. As a result, this may limit the applicability of some of this study’s data and suggests that any similar future study should consider the use of Intergrowth 21st or equivalent birthweight-based growth chart. However, we believe the study’s results have particular relevance because the study cohort of moderately preterm and late preterm neonates, represents the great majority of preterm infants hospitalised after birth. 

## 5. Conclusions

Our study has shown that a feeding protocol change that commenced nutrient supplementation at half enteral feed volumes (80 mL/kg/day) compared to full enteral feed volumes (160 mL/kg/day), resulted in predominately breastmilk fed infants commencing supplementation around 5 days earlier. This earlier supplementation was associated with significant clinically important benefits in reducing neonatal jaundice such as lowered levels of hyperbilirubinemia and a reduction in the frequency and extent of phototherapy. There were also additional benefits such as improved growth outcomes, reduced feeding intolerance and an almost 3-day reduction in LOS. We speculate that the improved jaundice outcomes are not related to any change in the dynamics of bilirubin liver production and conjugation, but due to mechanisms promoting more efficient elimination. The earlier nutrient supplementation occurred well before peak jaundice occurred, allowing time for fortification components to induce intestinal UGT1A1 (opposing the effects of β-glucuronidase) as well as promoting bowel motions—thus enhancing bilirubin excretion and elimination and reducing the enterohepatic recirculation of bilirubin.

## Figures and Tables

**Table 1:** Infant baseline characteristics.

	F80 (*n* = 105)	F160 (*n* = 110)	*p* Value
Gestational Age (weeks), mean ± sd.	34.6 ± 1.1	34.3 ± 1.1	0.107
Gender (male)	51 (49)	57 (52)	0.683
Birth weight (g), mean ± sd.	2134 ± 357	2076 ± 349	0.881
Birth weight (Z-score), mean ± sd.	−0.58 ± 0.93	−0.55 ± 0.88	0.761
Days to start supplement, mean ± sd	2.6 ± 0.8	7.4 ± 1.7	<0.0001
Reason for prematurity			
PPROM	32 (30)	40 (36)	0.388
Preeclampsia	13 (12)	14 (13)	1
APH	6 (6)	9 (8)	0.595
SGA	29 (28)	19 (170)	0.074
Multiples	7 (7)	8 (7)	1
Non-reassuring CTG	15 (14)	11 (10)	0.405
Mode of delivery			
Normal vaginal birth	41 (39)	45 (41)	0.889
Instrumental delivery	8 (8)	5 (5)	0.345
Elective caesarean	15 (14)	11 (10)	0.225
Emergency caesarean	41 (39)	48 (44)	0.293

*n* (%) unless otherwise stated. Student’s *t*-test for birth weight (Z-score) and gestational age. Mann–Whitney test for birthweight (g). Chi-square test for categorical variables. PPROM; preterm premature rupture of membrane. APH; antepartum hemorrhage. SGA; small for gestational age. CTG; cardiotocography. Z-score derived from Fenton growth charts for preterm infants.

**Table 2:** Maternal baseline characteristics.

	F80 (*n* = 105)	F160 (*n* = 110)	*p* Value
Age (years), mean ± sd	29.6 ± 5.3	30.5 ± 5.5	0.237
Region of Birth			0.394
Australia or New Zealand	59 (56)	48 (44)	
Middle East	9 (9)	12 (11)	
South Asia	24 (23)	36 (33)	
Others	11 (10)	12 (11)	
Language			0.449
English	92 (88)	92 (84)	
Arabic	9 (9)	7 (6)	
Others	4 (4)	10 (9)	
Antenatal Complications			
Pre-eclampsia	18 (17)	20 (18)	0.722
GDM	30 (29)	30 (27)	0.664
GBS positive	6 (6)	3 (3)	0.510

*n* (%). Student’s *t*-test for maternal age, Chi-square test for region of birth, language and antenatal complications. GDM; gestational diabetes. GBS; group B streptococcus.

**Table 3:** Anthropometric findings.

	F80 (*n* = 105)	F160 (*n* = 110)	*p* Value
Maximum weight loss (g), mean ± sd	108 ± 8	118 ± 6	0.072
Maximum weight loss as % birth weight	5%	6%	0.034
Days to regain birth weight, mean ± sd	10.0 ± 0.3	11.5 ± 0.3	<0.0001
Weight gain: Δ Z-score at 10 days, mean ± sd	−0.70 ± 0.03	−0.79 ± 0.03	0.01
Weight gain: Δ Z-score at discharge, mean ± sd	−0.64 ± 0.03	−0.68 ± 0.04	0.14
Discharge weight (g), mean ± sd	2348 ± 293	2359 ± 273	0.785

Chi-square test for maximum weight loss as % birth weight. Student’s *t*-test for remaining variables. Z-score derived from Fenton growth charts for preterm infants.

**Table 4:** Jaundice Outcomes.

	F80 (*n* = 105)	F160 (*n* = 110)	*p* Value
Phototherapy Extent			
One Light	9 (9)	24 (22)	0.008
Two Lights	19 (18)	17 (15)	0.7153
Three Lights	13 (12)	29 (26)	0.0103
Median Dose (number of lights)	2	2	
Maximum S. Bilrubin Value			
TSBR (Mean ± sd); µmol/l	196 ± 46	228 ± 52	0.0003
USBR (Mean ± sd); µmol/l	184 ± 44	212 ± 50	0.0008
CSBR (Mean ± sd); µmol/l	12 ± 4	16 ± 5	<0.0001
% CSBR of TSBR (Mean ± sd)	6.2 ± 1.6	7.0 ± 2.8	0.0694
Days to Maximum TSBR	4.8 ± 1.8	5.1 ± 2.0	0.4298

*n* (%). Chi-square test for Phototherapy Extent. Student’s *t*-test for Bilirubin values. TSBR—total serum bilirubin. USBR—unconjugated bilirubin CSBR—and conjugated bilirubin. There was no incidence of conjugated hyperbilirubinemia (as defined by CSBR being at a level of more than 20% of TSBR) in either group.

**Table 5:** Adverse clinical outcomes.

	F80 (*n* = 105)	F160 (*n* = 110)	*p* Value
Feeding Intolerance	28 (26.0)	49(45.2)	0.007
Presumed Sepsis	41 (48)	67 (60)	0.693
RDS	16 (15)	27 (24)	0.882
Hypoglycaemia	27 (25)	30 (27)	0.818
NEC	0 (0)	0 (0)	1
LOS in SCN (days), mean ± sd	16.0 ± 0.6	18.8 ± 0.7	0.036

*n* (%). Chi-square test for all variables except LOS which was student’s *t*-test. NEC—necrotising enterocolitis. LOS—Length of stay. RDS—Respiratory Distress Syndrome. SCN—Special Care Nursery. Hypoglycaemia defined by plasma glucose < 2.6 mmol/L.
